# Comparative efficacy of materials used in patients undergoing pulpotomy or direct pulp capping in carious teeth: A systematic review and meta‐analysis

**DOI:** 10.1002/cre2.767

**Published:** 2023-09-14

**Authors:** Athanasios Fasoulas, Georgios Keratiotis, Loukia Spineli, Nikos Pandis, Mieke A. A. De Bruyne, Roeland J.G. De Moor, Maarten A. Meire

**Affiliations:** ^1^ Department of Oral Health Sciences, Section of Endodontology Ghent University Ghent Belgium; ^2^ Midwifery Research and Education Unit Hannover Medical School Hannover Germany; ^3^ Department of Orthodontics and Dentofacial Orthopaedics University of Bern Bern Switzerland

**Keywords:** Biodentine, calcium hydroxide, direct pulp capping, MTA, pulpotomy, vital pulp treatment

## Abstract

**Objectives:**

Different materials have been used for capping the pulp after exposure during caries removal in permanent teeth. The purpose of this study was to collate and analyze all pertinent evidence from randomized controlled trials (RCTs) on different materials used in patients undergoing pulpotomy or direct pulp capping in carious teeth.

**Materials and Methods:**

Trials comparing two or more capping agents used for direct pulp capping (DPC) or pulpotomy were considered eligible. An electronic search of four databases and two clinical trial registries was carried out up to February 28, 2021 using a search strategy properly adapted to the PICO framework. Screening, data extraction, and risk of bias (RoB) assessment of primary studies were performed in duplicate and independently. The primary outcome was clinical and radiological success; secondary outcomes included continued root formation, tooth discoloration, and dentin bridge formation.

**Results:**

21 RCTs were included in the study. The RoB assessment indicated a moderate risk among the studies. Due to significant clinical and statistical heterogeneity among the studies, performing network meta‐analysis (NMA) was not possible. An ad hoc subgroup analysis revealed strong evidence of a higher success of DPC with Mineral Trioxide Aggregate (MTA) compared to calcium hydroxide (CH) (odds ratio [OR] = 3.10, 95% confidence interval [CI]: 1.66−5.79). MTA performed better than CH in pulp capping (both DPC and pulpotomy) of mature compared to immature teeth (OR = 3.34, 95% CI: 1.81−6.17). The GRADE assessment revealed moderate strength of evidence for DPC and mature teeth, and low to very low strength of evidence for the remaining subgroups.

**Conclusions:**

Considerable clinical and statistical heterogeneity among the trials did not allow NMA. The ad hoc subgroup analysis indicated that the clinical and radiographic success of MTA was higher than that of CH but only in mature teeth and DPC cases where the strength of evidence was moderate. PROSPERO Registration: number CRD42020127239.

## 1 INTRODUCTION

Due to poor expected outcomes, clinicians frequently neglect vital pulp treatment (VPT) as a treatment option in case of a cariously exposed pulp, and root canal treatment is mostly seen as the only treatment option (Horsted et al., 1985). Recent clinical studies and systematic reviews, however, challenge this belief, as they highlight the success of VPT, even in cases of mature teeth with pulpal inflammation (Aguilar & Linsuwanont, 2011; Paula et al., 2018). Research has demonstrated the reparative potential of the dentine‐pulp complex and the presence of healthy tissue near inflamed or necrotic pulp (Cooper et al., 2014; Simon et al., 2011; Smith et al., 2016). Last but not least, the use of magnification and biocompatible materials improve VPT outcomes (Bogen et al., 2008; European Society of Endodontology developed et al., 2019; Marques et al., 2015).

By preserving pulp vitality, the tooth retains its sensibility and mechanoreception, as well as the ability of dentinogenesis and root development (Bjorndal et al., 2019; Schwendicke, 2019). This is in contrast to a pulpectomy procedure, where the entire pulp is removed at the cost of more extensive hard tissue loss, negatively affecting tooth integrity (Wolters et al., 2017).

The position statement on the management of deep caries and the exposed pulp of the European Society of Endodontology (Duncan et al., 2019) presents treatment options.

The radiographic distinction between deep and extremely deep carious lesions is made. In case of deep caries with clinical manifestations of reversible pulpitis, selective or stepwise excavation may be the best intervention, avoiding pulp exposure (Duncan et al., 2019; Schwendicke et al., 2021). When pulp exposure is yet the case or unavoidable in the presence of extremely deep caries, alternative treatment modalities such as direct pulp capping (DPC), and partial or full pulpotomy may be considered. Deep caries with reversible pulpitis may benefit from selective excavation, while pulp exposure calls for DPC or pulpotomy (Duncan et al., 2019; Wolters et al., 2017). For the optimal success of VPT, it is essential to adhere to an “optimized protocol”. This involves strict aseptic measures (removal of plaque and calculus before tooth isolation, field disinfection, use of sterile rotary instruments, and use of disinfecting solutions for pulp hemostasis), and the use of magnification in the steps of caries removal, pulp amputation, and pulp capping with a biocompatible material.

Many materials to cap the exposed pulp have already been compared head‐to‐head in studies of different designs (Aguilar & Linsuwanont, 2011). The importance of randomized controlled trials to examine a causal relationship between an intervention and an outcome has been repeatedly emphasized (Schulz et al., 2010). A plethora of small‐size trials has been conducted to date comparing different capping materials mainly in terms of clinical and radiographic success. Subsequent systematic reviews (Cushley et al. 2019, 2021; Didilescu et al., 2018; Li et al., 2019) synthesized the aforementioned studies by means of pairwise meta‐analysis. Capping agents were, thus, compared in pairs but never all together. Consequently, the clinical question, “which material performs the best,” remains yet unanswered. Network meta‐analysis (NMA) is a statistical methodology for direct and indirect comparison of different interventions by forming a network (Salanti, 2012). Studies that compare at least two of these interventions—for example, pulp capping materials—may contribute to the network. By analyzing trials that share a common comparator intervention, indirect evidence can be drawn for comparisons that were not directly studied (Bucher et al., 1997).

To our knowledge, to date, there is no systematic review comparing all available capping materials. Furthermore, previous studies did not adequately focus on secondary outcomes such as tooth discoloration, root formation, and pulp sensibility. The aim of the present study is to answer the following clinical question: “In patients with carious exposure, which material, when used as a capping agent in cases of DPC or pulpotomy, results in the highest clinical and radiographic success, and best outcome with regard to discoloration, root development, tooth sensibility, and bridge formation?”.

## 2 MATERIALS AND METHODS

### 2.1 Protocol registration and eligibility criteria

The protocol of this study was registered before the commencement of the study in the PROSPERO database (https://www.crd.york.ac.uk/prospero) with registration number CRD42020127239. Reporting was conducted in line with the PRISMA extension for NMA guidelines (Hutton et al., 2015).

The eligibility criteria for study selection were the following:

Population: We included Randomized controlled trials (RCTs) of patients with carious pulp exposure in one or more of their permanent teeth diagnosed with reversible or irreversible pulpitis and treated with either DPC, partial pulpotomy, or full pulpotomy.

Intervention/Comparator: Following Chaimani et al. (Chaimani et al., 2017), we considered all retrieved interventions to be of interest for analysis, and we did not distinguish between I and C since an agent may be the control in one trial, but the experimental in another. All available biomaterials used to cap the amputated pulp tissue were considered (e.g., Calcium hydroxide (CH), Mineral Trioxide Aggregate (MTA), Biodentine, and other agents).

Outcomes: The primary outcome was the composite outcome of both clinical and radiographic success of pulp capping after at least 6 months of follow‐up determined by

- absence of spontaneous pain, lingering/heightened reaction on thermal stimuli and sensitivity/pain upon percussion/palpation, or other soft tissue signs (swelling, sinus tract)
- absence of radiographic evidence of periapical changes indicative of apical periodontitis

Secondary outcomes:

- Tooth sensibility
- Continuation of root formation
- Bridge formation (radiographically assessed)
- Discoloration

Duration: A study to be included had to have a minimum of 6 months follow‐up.

Lumping of interventions: In the primary analysis, different CH formulations (e.g., Dycal, Life, and pure CH) and different MTA products were considered together.

Language: No language restrictions were applied. Non‐English reviews were first translated into English, and if eligible for inclusion, full data extraction was performed.

Date of publication: There was no restriction in terms of the date of publication.

### 2.2 Search strategy, study selection, and data extraction

A search strategy using specific keywords and Mesh terms combined with appropriate Boolean operators was drafted. The search strategy applied in PubMed is displayed in Table 1. The electronic search was conducted within published and unpublished research, across 4 databases (Medline via PubMed, Embase, Scopus, and Web of Science), two clinical trial registers (Clinicaltrials.gov and WHO [ICTRP]), and two gray literature databases (GreyLit and OpenGrey) (Table 2). These databases were searched until 28/2/2021. Furthermore, the reference list of all included articles was screened by two reviewers (A. F. and G. K.).

**Table 1:** Search strategy template applied in PubMed.

Research question (e.g., PICO format):		
NAME OF DATABASE (interface): MEDLINE via PubMed		
Concept	Line number	Search strategy
Concept 1:	1	pulpotom*[tw] OR “pulpotomy”[mh] OR “vital pulp treatment”[tw] OR “vital pulp therapy”[tw] OR “pulp amputation”[tw] OR capping [tw] OR “dental pulp capping”[mh] OR pulp exposure[tw] OR “dental pulp exposure”[mh] OR pulpitis [tw] OR pulpitis [mh] OR dental caries[tw] OR “dental caries”[mh] OR dental decay[tw] OR tooth decay[tw] OR carious dentin*[tw]
Concept 2:	2	calcium hydroxide[tw] OR “calcium hydroxide” [mh] OR mta[tw] OR mta‐angelus[tw] OR endosequence[tw] OR totalfill [tw] OR “mineral trioxide aggregate”[tw] OR “Portland cement”[tw] OR “calcium silicate”[tw] OR “tricalcium silicate”[tw] OR “calcium enriched mixture” [tw] OR “cem cement”[tw] OR endocem[tw] OR biodentine [tw] OR formocresol [tw] OR formocresols [mh] OR “ferric sulfate”[tw] OR emdogain [tw] OR “enamel matrix derivative”[tw] OR “Platelet‐rich Fibrin”[tw] OR l‐PRF[tw] OR “zinc oxide eugenol”[tw] OR “Zinc Oxide‐Eugenol Cement”[mh] OR ZOE[tw]
Filter/search block:	3	randomized controlled trial [pt] OR multicenter study [pt] OR controlled clinical trial [pt] OR clinical study [pt] OR clinical trial [pt] OR clinical trials as topic [mh] OR drug therapy[sh] OR “drug therapy”[mh] OR “prospective stud*” [tw] OR random* [tw] OR placebo* [tw] OR placebos[mh] OR trial* [tw] OR group* [tw] OR blind* [tw] OR allocat* [tw] OR “factorial design” [tw] OR “factorial trial” [tw] OR “multicenter study” [tw] OR “multicentre study” [tw] OR rct [tw] NOT (animals [mh] NOT humans [mh])
Combination of concepts	4	1 AND 2 AND 3

**Table 2:** Dates of coverage in electronic databases searched.

Name	Dates of coverage
Electronic databases	
PubMed (pubmed.gov)	From inception to 28/2/21
Embase (embase.com)	From inception to 28/2/21
Scopus (scopus.com)	From inception to 28/2/21
Web of Science (webofknowledge.com)	From inception to 28/2/21
CENTRAL (cochranelibrary.com/central)	From inception to 28/2/21
Trial registries	
Clinical Trials (clinicaltrials.gov)	From inception to 28/2/21
WHO (ICTRP)	From inception to 28/2/21
Gray literature databases	
GreyLit (greylit.org)	From inception to 28/2/21
OpenGrey (opengrey.eu)	From inception to 28/2/21

The records retrieved from the electronic search were introduced into a reference manager (EndNote X9) and deduplicated following the strategy suggested by Bramer et al. (Bramer et al., 2016). The remaining records were introduced into an electronic web application designed for the screening procedure in systematic reviews (rayyan.qcri.org). Two reviewers (A. F. and G. K.) independently and in duplicate screened all titles and abstracts against the eligibility criteria. Liberal acceleration was employed; thus, in case of disagreement, the record was included for further full‐text screening. The full text of the included studies was assessed for eligibility by the same two reviewers in duplicate and independently (Edwards et al., 2002). Any disagreement was resolved by discussion with a third reviewer (M. M.) (Bomhof‐Roordink et al., 2019). Two independent reviewers (A. F. and G. K.) extracted in duplicate the predetermined data items from all included studies (Buscemi et al., 2006). A predefined data extraction sheet was drafted in Excel (Microsoft Office 365) and is presented in Supporting Information Material (Supporting Information: Table S1). Data extracted included trial‐related information (e.g., type of trial, number of patients randomized, type of randomization, blinding, missing participants, number of centers, period of follow‐up, and so on), baseline characteristics of the participants recruited (e.g., age of patients, type of teeth, root development status, and pulpal and periapical diagnosis), clinical protocol information (type of treatment, size of exposure, use of rubber dam (RD), disinfection protocol, capping agents used, use of magnification, and type of operator), and outcomes (e.g., number of patients missing, number of patients in each group that failed, and number of patients in each group with discoloration). In case of missing information, the corresponding authors of the studies were contacted using the mail address provided in the paper.

### 2.3 Risk of bias (RoB)

Two reviewers (A. F. and G. K.) assessed independently and in duplicate the RoB of individual studies according to the revised RoB 2 (Sterne et al., 2019). A template was used to assess five different domains for each study. Supporting information justifying the decision of individual judgments was recorded. An overall assessment of “low,” “some concerns,” or “high” was reached for every study. In case of disagreement, a discussion with a third reviewer (N. P.) was performed to reach a consensus.

### 2.4 Εvaluation of the transitivity assumption

The transitivity assumption states that important (trial‐level) characteristics that act as effect modifiers should be similarly distributed across the observed comparisons (Jansen et al., 2012; Jansen & Naci, 2013). Each trial should have reported every characteristic of interest to allow for the evaluation of this assumption. Comparisons with missing characteristics or characteristics that are unclearly reported make it difficult to defend the transitivity assumption. We used a stacked barplot to explore the frequency of the following characteristics across the observed pairwise comparisons in the network: pulpal diagnosis, type of teeth included, root maturation, and treatment performed (DPC, partial pulpotomy, and full pulpotomy). When the levels of a characteristic appear in a similar frequency across the observed comparisons, this indicates possible transitivity for that characteristic.

### 2.5 Certainty of evidence

Two reviewers (A. F. and G. K.) independently assessed the certainty of evidence using the GRADEpro GDT software to prepare and present the “Summary of findings” table.

### 2.6 Analysis performed

The log odds ratio (OR) and the accompanying 95% confidence interval (CI) were calculated for each trial while adjusting for the missing participant outcome data (MOD) in each arm, where available (Methods section in Supporting Information Material). A continuity correction of 0.5 was used to adjust the number of events in each arm of those trials with zero events in at least one arm. Results are presented in the OR scale.

For the split‐mouth studies, a correlation of 0.5 was used to obtain the statistically correct standard error of log OR. For the cluster RCTs, the log OR and standard error were adjusted for different values of intra‐cluster correlation (in the range from 0 to 0.5 and increment of 0.025) and average cluster size of two (Pandis et al., 2013). An intra‐cluster correlation of 0.5 was considered for the primary analysis.

Due to substantial clinical and statistical heterogeneity, a pairwise meta‐analysis for comparisons with at least two trials was not performed. Instead, a panel of forest plots was created for each observed comparison and outcome, and the three fully observed characteristics were illustrated using different line‐color, line‐shape, and point‐shape, respectively. The R‐package *ggplot2* was used to create the panel of forest plots and the barplot for the visual evaluation of the transitivity assumption (Wickham, 2009). The R‐package *pcnetmeta* was used to draw the network plots (Lin et al., 2017). In the Methods section in Supporting Information Material, detailed information is provided on the analyses planned but not considered and the analyses employed to obtain the results.

### 2.7 Ad hoc analyses

A subgroup analysis was performed for the comparison of CH versus MTA, separately, for the treatment performed (i.e., DPC and FP/PP) and root maturation (i.e., mature, immature, and mixed). CH versus MTA was chosen primarily for the low RoB in most trials and second for having the most trials in the network. The choice of the subgroup was predefined in the protocol of the study where treatment performed (Aguilar & Linsuwanont, 2011) and root development status (Chen et al., 2019) were considered as potential effect modifiers. An inverse‐variance random‐effects meta‐analysis was performed using the Knapp‐Hartung adjustment (Knapp & Hartung, 2003) of the summary standard error and the restricted maximum likelihood estimator for the between‐trial variance parameter ($\tau^{2}$) (Raudenbush, 2009). As input data, the log OR and standard error after adjusting for MOD were used in every trial. Forest plots were created to present the subgroup analyses, separately. The 95% prediction intervals are also presented in each subgroup. The subgroup analyses were performed using the R‐package *metafor (*Viechtbauer, 2020 *)* (statistical software R, version 4.0.3).

## 3 RESULTS

### 3.1 Study selection

**Figure 1:**
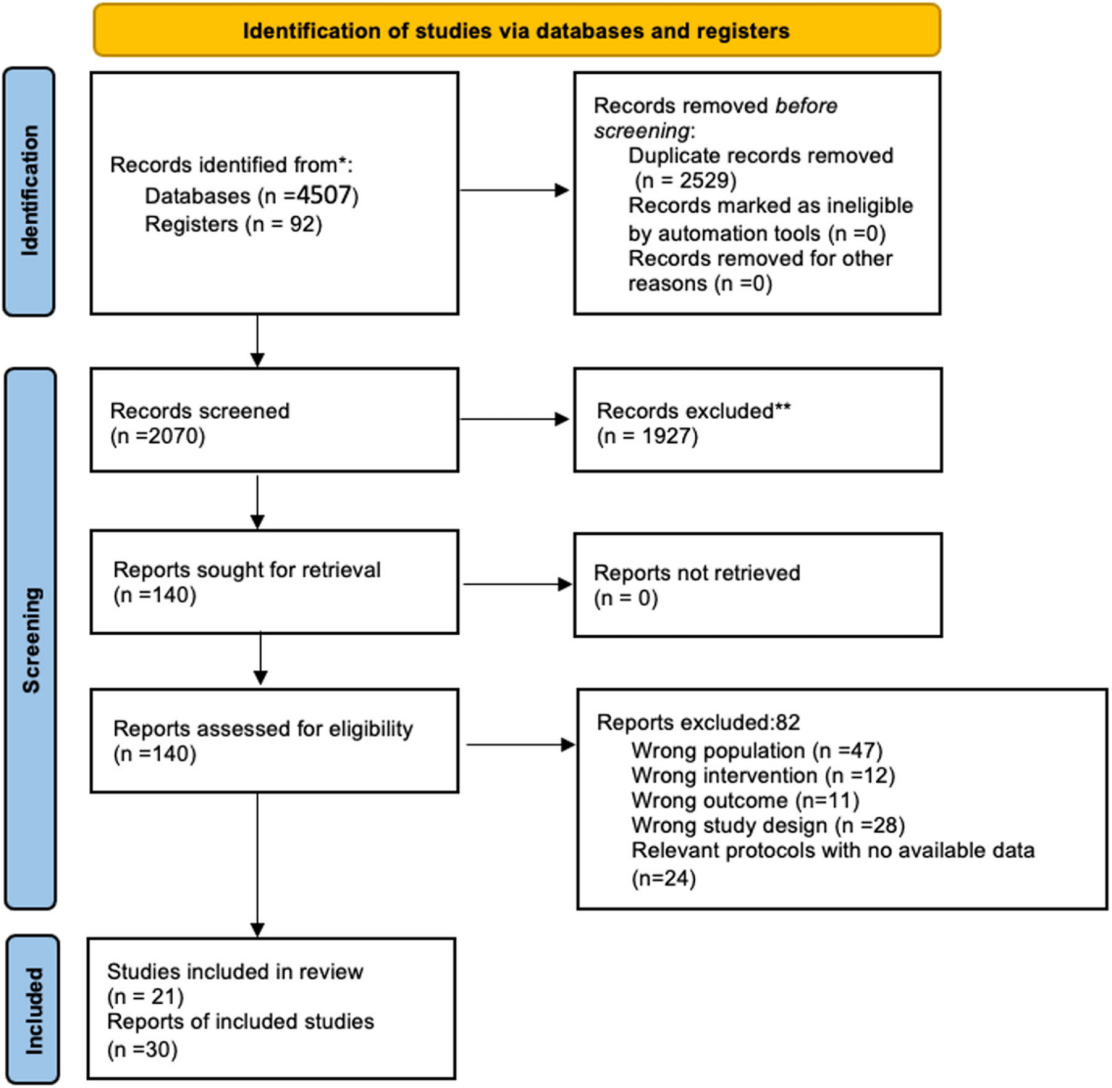
PRISMA flowchart of literature search and screening.

Four thousand five hundred and seven records were retrieved from the electronic databases and another 92 from Clinicaltrials.gov. The search in gray literature websites produced no results. A total of 4599 records were entered in EndNote, and after removing the duplicates, 2070 records were entered into Rayyan. After screening titles and abstracts, 140 records we considered relevant, and their full texts were retrieved to assess their eligibility. Finally, 54 records in total met the eligibility criteria. Figure 1 shows the flow of the study. We employed Gwet's AC1 (Wongpakaran et al., 2013) to estimate the interobserver's agreement using the rel package (Lo Martire, 2017) in the R studio (statistical software R, version 4.0.3). The Gwet's AC1 was equal to 0.86 (95% CI: 0.77−0.94, standard error [SE] = 0.04), indicating a very good level of agreement between the two reviewers that performed screening. Thirty records representing 21 different studies (Alawwad et al., 2020; Asgary et al., 2017; Awawdeh et al., 2018; Brizuela et al., 2017; Cengiz & Yilmaz, 2016; Chailertvanitkul et al., 2014; El‐Meligy & Avery, 2006; Eppa et al., 2018; Keswani et al., 2014; Kumar et al., 2016; Kundzina et al., 2017; Nosrat et al., 2013; Ozgur et al., 2017; Parinyaprom et al., 2018; Peskersoy et al., 2021; Qudeimat et al., 2007; Suhag et al., 2019; Taha & Khazali, 2017; Uesrichai et al., 2019; Vu et al., 2020; Wei et al., 2010) were further introduced for data extraction. The remaining 24 records were protocols of potentially eligible studies. Contact with the authors did not result in any extra information about ongoing trials.

### 3.2 Study characteristics

**Figure 2:**
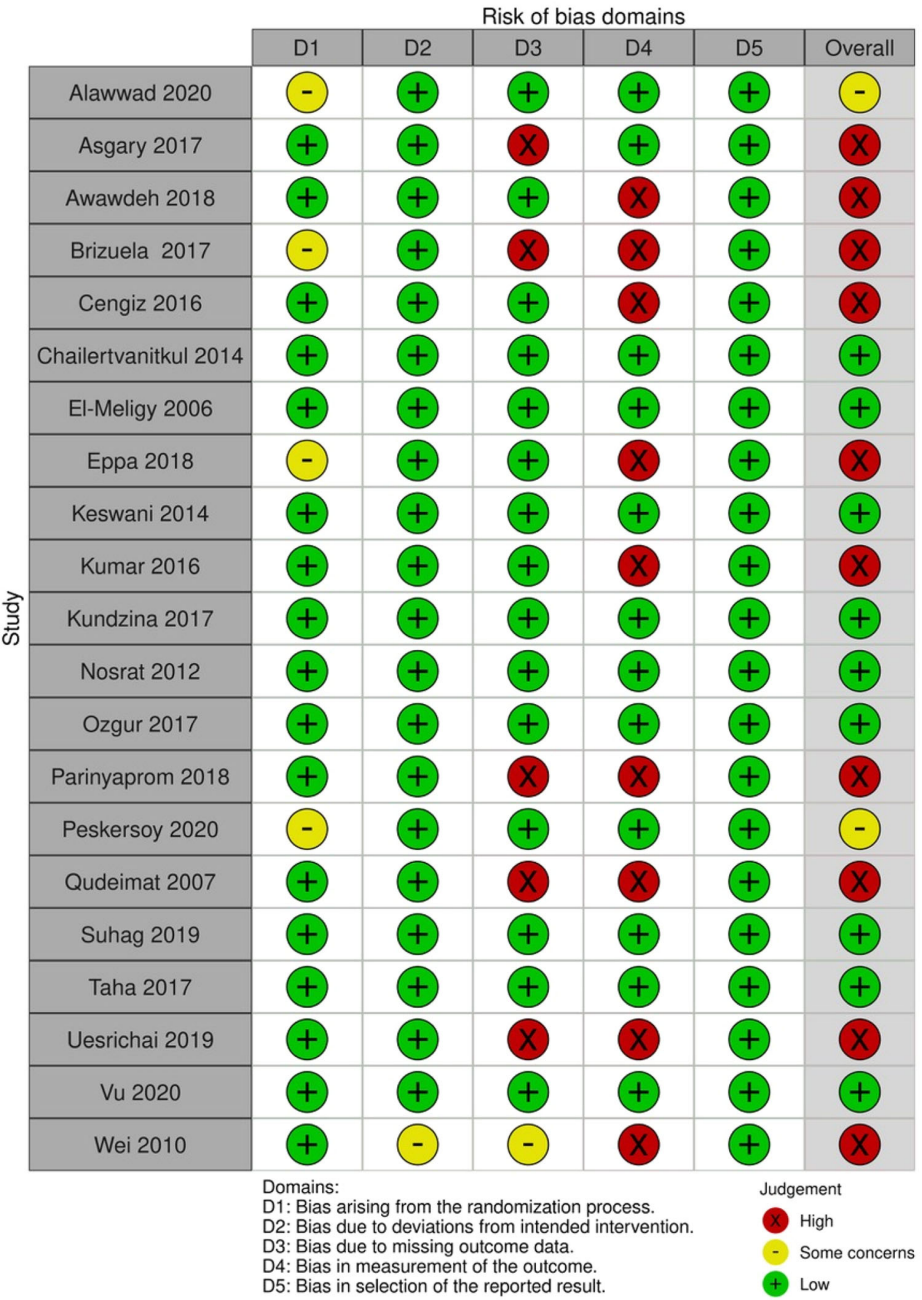
Network plot of the different capping materials for the primary outcome clinical and radiographical success. Each intervention is indicated by a node and each comparison by a link between two nodes. The size of the nodes and the thickness of the links are weighted by the number of trials investigating the corresponding interventions and comparisons, respectively. Colored templates were used to indicate multi‐arm trials. Acem, Acemannan; AR, Abscess remedy; BD, Biodentine; CEM, Calcium enriched mixture; CH, calcium hydroxide; NHA, nano‐hydroxyapatite; PRF, platelet‐rich fibrin; TAP, triple antibiotic paste.

**Figure 3:**
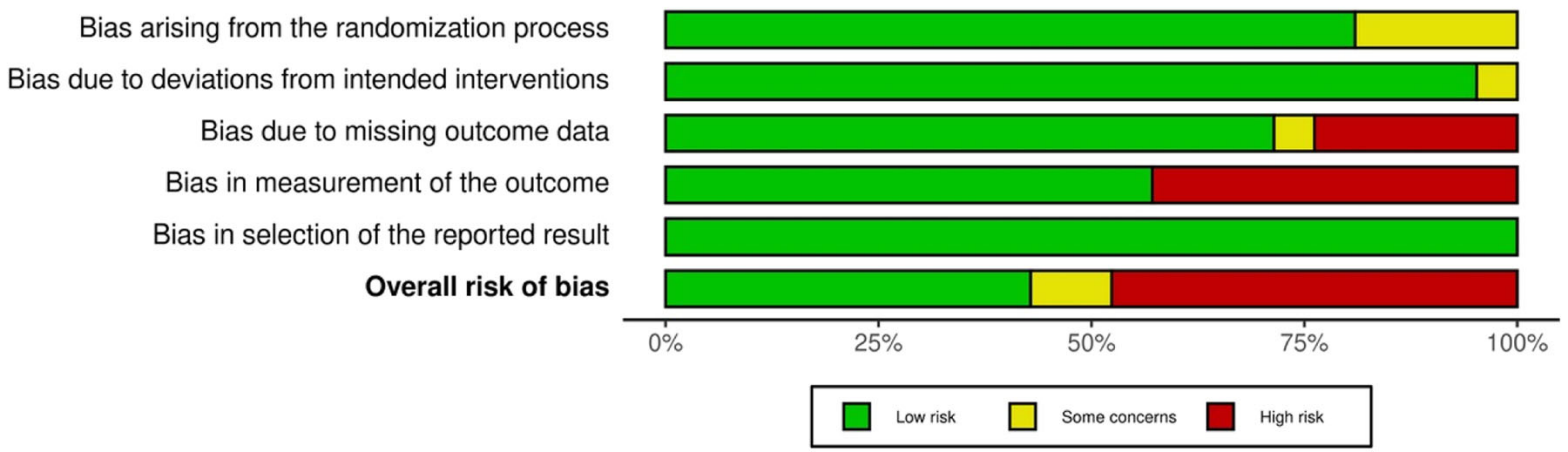
The network plot for the secondary outcome root development. Each intervention is indicated by a node and each comparison by a link between two nodes. The size of the nodes and the thickness of the links are weighted by the number of trials investigating the corresponding interventions and comparisons, respectively. Colored templates were used to indicate multiarm trials.

**Figure 4:**
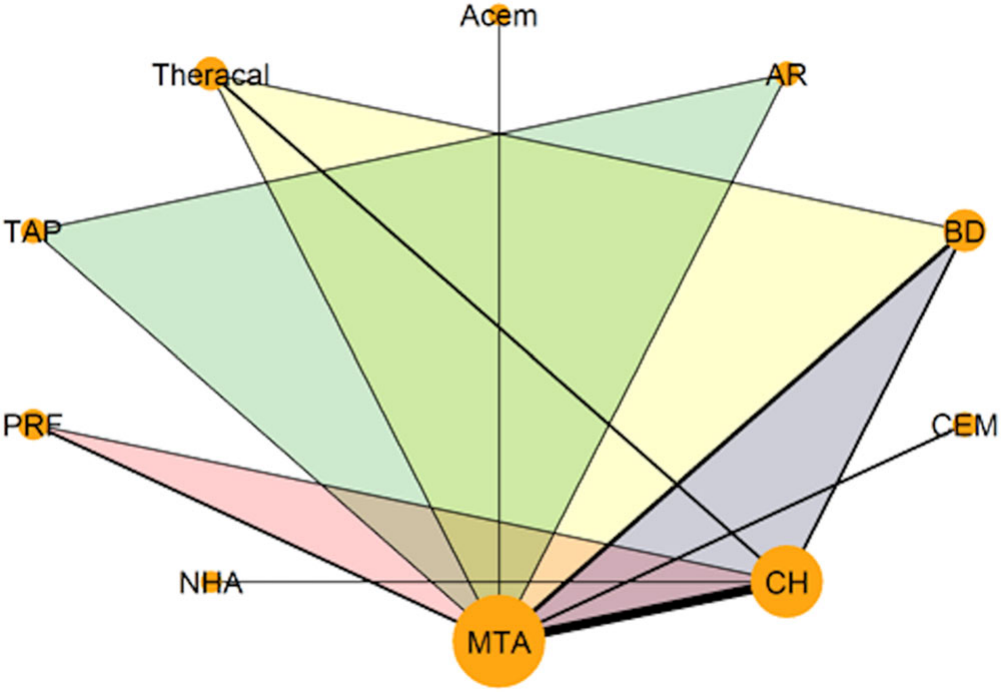
Network plot for the secondary outcome tooth discoloration. Each intervention is indicated by a node and each comparison by a link between two nodes. The size of the nodes and the thickness of the links are weighted by the number of trials investigating the corresponding interventions and comparisons, respectively.

**Figure 5:**
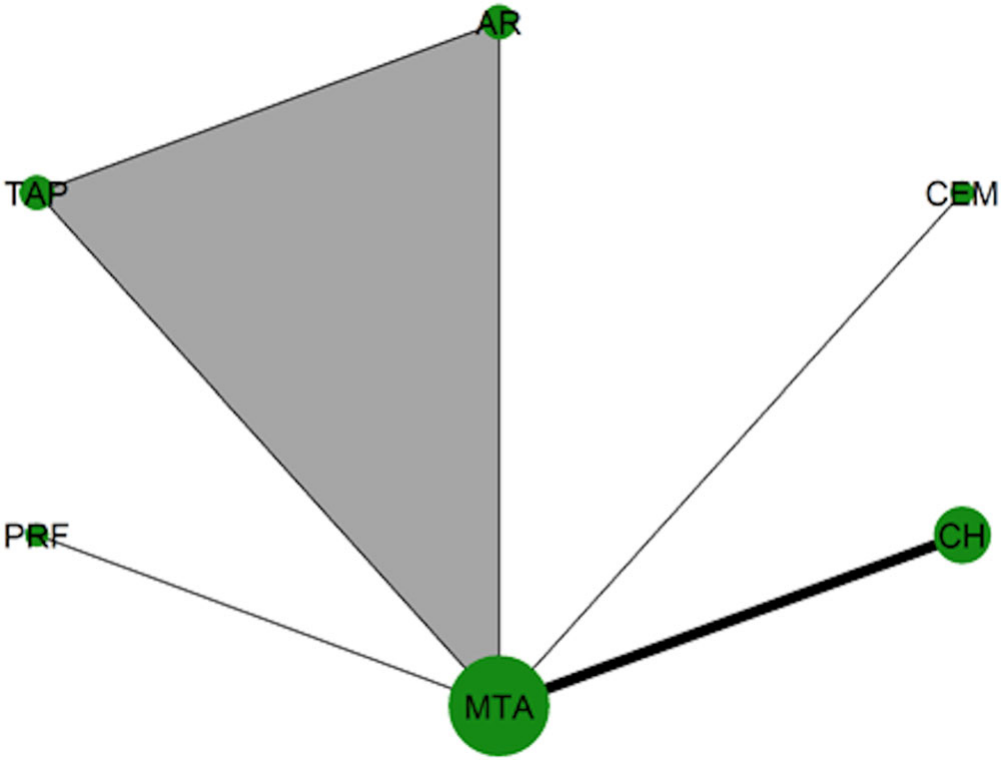
Network plot for the secondary outcome bridge formation. Each intervention is indicated by a node and each comparison by a link between two nodes. The size of the nodes and the thickness of the links are weighted by the number of trials investigating the corresponding interventions and comparisons, respectively.

Table 3 shows the characteristics of the included studies. The primary outcome has been investigated in 21 trials (17 two‐arm, three three‐arm trials, and one four‐arm trial) that form a complex network of 10 interventions (Figure 2). Two of the studies (Cengiz & Yilmaz, 2016; Özgür et al., 2017) used stratified randomization for the hemostasis/disinfection protocol (see Table 4); thus, different strata were treated like different studies in the meta‐analysis performed. Two out of the five arms of Peskersoy et al. (2021). represented different CH agents, so they were lumped together. Only 31% of the possible comparisons have been informed by at least one trial. MTA and CH are the most frequently studied interventions (20 and 14 trials, respectively), followed by BD with five trials, Theracal and PRF with three trials, and CEM and PRF with two trials. CH versus MTA is the most prevalent comparison (11 trials), followed by BD versus MTA (five trials). CEM versus MTA and CH versus BD were investigated in 2 trials each. The network for the secondary outcome “root development” (Figure 3) consists of less than half the trials (six two‐arm trials and one three‐arm trial) and comparisons observed in the primary outcome network. The remaining secondary outcomes refer to open triangle networks (Figure 4 and 5) with one comparison informed by one trial only.

**Table 3:** Study design and important trial information.

First author	Type of RCT	Pulpal diagnosis	Type of teeth included	Root maturation	Treatment performed	Mean/median age (SD)	Group 1 (No teeth randomized)	Group 2 (No teeth randomized)	Group 3 (No teeth randomized)	Group 4 (No teeth randomized)	Group 5 (No teeth randomized)	Patient randomized in case of cluster effect
Alawwad et al. (2020)	Randomized parallel group trial	Irreversible pulpitis	Molars	Immature	FP	NA	PD White MTA (12)	PRF (12)	NA	NA	NA	20 patients in total
Asgary et al. (2017)	Randomized parallel group trial	Irreversible pulpitis	Molars	Mature	FP	NA	Pro Root MTA(208)	CEM (205)	NA	NA	NA	‐
Awawdeh et al. (2018)	Randomized parallel group trial	Reversible pulpitis/Irreversible pulpitis	All type of teeth	Mature	DPC, FP	μ = 32.5	wMTA Angelus (34)	Biodentine (34)	NA	NA	NA	‐
Brizuela et al. (2017)	Randomized parallel group trial	Normal pulp/Reversible pulpitis	Molars	Mixed	DPC	μ = 11.3 (2.44), δ = 11	CH powder (53)	Pro Root MTA (56)	Biodentine (60)	NA	NA	‐
Cengiz (2016a)	Randomized parallel group trial	NA	Premolars and molars	Mature	DPC	μ = 28	Dycal (15)	Theracal (15)	NA	NA	NA	‐
Cengiz (2016b)	Randomized parallel group trial	NA	Premolars and molars	Mature	DPC	μ = 28	Dycal (15)	Theracal (15)	NA	NA	NA	‐
Chailertvanitkul et al. (2014)	Randomized parallel group trial	Reversible pulpitis	Molars	Mixed	PP	NA	Dycal (40)	Pro Root MTA (44)	NA	NA	NA	40/40
El‐Meligy & Avery (2006)	Split‐mouth trial	NA	Premolars and molars	Immature	FP	NA	CH powder (13)	MTA (13)	NA	NA	NA	‐
Eppa et al. (2018)	Randomized parallel group trial	NA	NA	Immature	FP	NA	MTA (20)	TAP (20)	Abscess Remedy (20)	NA	NA	‐
Keswani et al. (2014)	Randomized parallel group trial	NA	Molars	Immature	FP	NA	MTA (31)	PRF (31)	NA	NA	NA	‐
Kumar et al. (2016)	Randomized parallel group trial	Irreversible pulpitis	Molars	Mature	FP	NA	CH powder (18)	Pro Root MTA (19)	PRF (17)	NA	NA	‐
Kundzina et al. (2017)	Randomized parallel group trial	NA	Molars	Mature	DPC	NA	Pro Root MTA (33)	Dycal (37)	NA	NA	NA	‐
Nosrat (2013)	Randomized parallel group trial	NA	Molars	Immature	FP	NA	Pro Root MTA (25)	CEM (26)	NA	NA	NA	‐
Ozgur et al. (2017)	Randomized parallel group trial	NA	Molars	Immature	PP	NA	Pro Root MTA (20)	CH powder (20)	NA	NA	NA	‐
Ozgur et al. (2017)	Randomized parallel group trial	NA	Molars	Immature	PP	NA	Pro Root MTA (20)	CH powder (20)	NA	NA	NA	‐
Parinyaprom et al. (2018)	Randomized parallel group trial	Normal pulp/Reversible pulpitis/Irreversible pulpitis	Molars	Mixed	DPC	μ = 10 (2)	Pro Root MTA (30)	Biodentine (29)	NA	NA	30	‐
Peskersoy et al. (2021)	Randomized parallel group trial	Reversible pulpitis	Molars	Mature	DPC	NA	Dycal (106)	Calcihyd (105)	Theracal (105)	Biodentine (105)	Bio MTA plus (105)	226 patients in total
Qudeimat et al. (2007)	Randomized parallel group trial	NA	Molars	Mixed	PP	10.3	Dycal (23)	Pro Root MTA (28)	NA	NA	NA	‐
Suhag et al. (2019)	Randomized parallel group trial	Reversible pulpitis	Molars	Mature	DPC	NA	Pro Root MTA (27)	CH powder (29)	NA	NA	NA	‐
Taha & Khazali (2017)	Randomized parallel group trial	Irreversible pulpitis	Molars	Mature	PP	μ = 30.3 (4.8)???	Pro Root MTA (27)	CH powder (23)	NA	NA	NA	‐
Uesrichai et al. (2019)	Randomized parallel group trial	Irreversible pulpitis	Molars	Mixed	PP	μ = 10 (2.1)	Pro Root MTA (37)	Biodentine (32)	NA	NA	NA	‐
Vu et al. (2020)	Randomized parallel group trial	Reversible pulpitis	Molars	Immature	PP	NA	Pro Root MTA (14)	Acemannan (13)	NA	NA	NA	‐
Wei et al. (2010)	Randomized parallel group trial	NA	All type of teeth	Mature	DPC	NA	Dycal (54)	Nano‐hydroxyapatite (59)	NA	NA	NA	47/51

*Note*: The last column corresponds to the number of patients in trials where more than one tooth was included from one patient.

Abbreviations: DPC, Direct pulp capping; FP, Full pulpotomy; NA, Not available; PP, Partial pulpotomy; RCT, randomized controlled trial.

**Table 4:** Clinical characteristics of the trials.

First author	Size of exposure	Method of disinfection	Method of hemostasis	Hemostasis outcome (time)	Base	Type of coronal restoration	Type of operator	Number of different operators	Use of RD	Use of magnifying device
Alawwad et al. (2020)	NA	Sterile saline	Moist cotton pellets	10–15 min	IRM	Amalgam restorations	NA	NA	Yes	NA
Asgary et al. (2017)	NA	Sterile saline	Sterile physiological saline‐moistened cotton pellets	NA	No	Amalgam restorations	General dentists	23	Yes	NA
Awawdeh et al. (2018)	NA	NA	5% NaOCl moistened cotton pellets	≤6 min (if not then proceed to PP)	NA	53/68 CR 15/68 Amalgam	NA	NA	Yes	NA
Brizuela et al. (2017)	≤2 mm	NA	Sterile physiological saline‐moistened cotton pellets	≤10 min	RMGIC	CR	Postgraduate students	5	Yes	Loupes x3.5
Cengiz (2016)	0.5−1.5 mm	NA	Sterile physiological saline‐moistened cotton pellets	≤3 min	RMGIC	CR	Specialist in conservative dentistry	1	Yes	NA
Cengiz (2016)	0.5−1.5 mm	Er; Cr: YSGG laser	Er; Cr: YSGG laser	≤3 min	RMGIC	CR	Specialist in conservative dentistry	1	Yes	NA
Chailertvanitkul et al. (2014)	Up to 10 mm^2^	Irrigation with 2,5% NaOCl	2,5% NaOCl moistened cotton pellets	1−2 min	Tri‐cure GIC	Amalgam restorations	Master students in endo	2	Yes	NA
El‐Meligy and Avery (2006)	ΝΑ	NA	Saline on cotton pellet	NA	ZOE (CH)	Amalgam restorations	NA	NA	Yes	No
Eppa et al. (2018)	NA	NA	Saline on cotton pellet	≤5 min	RMGIC	SSC	NA	NA	Yes	NA
Keswani et al. (2014)	NA	NA	Saline on cotton pellet	≤5 min	ZOE (MTA)	Amalgam & SSC	NA	1	Yes	NA
Kumar et al. (2016)	NA	Sterile saline	NA	NA	RMGIC	CR	NA	1	Yes	NA
Kundzina et al. (2017)	NA	NA	0,5% NaOCl moistened cotton pellets	10 min	Fuji IX	CR	NA	6	Yes	NA
Nosrat (2013)	NA	NA	Saline on cotton pellet	10 min	GIC	NA	Endodontist	1	Yes	NA
Ozgur et al. (2017)	1−2 mm	NA	Saline 0.9% soacked pellets	5 min	GIC	CR	Pediatric dentist	1	Yes	NA
Ozgur et al. (2017)	1−2 mm	NA	2,5% NaOCl moistened cotton pellets	5 min	GIC	CR	Pediatric dentist	1	Yes	NA
Parinyaprom et al. (2018)	≤2.5 mm	Irrigation with 2,5% NaOCl	2,5% NaOCl moistened cotton pellets	≤10 min	MTA: RMGIC Biodentine: No	39/59 CR 1/59 Amalgam 15/59 SSC	Postgraduate students	8	Yes	NA
Peskersoy et al. (2021)	Up to 1 mm	Irrigation with 2,5% NaOCl	Saline on cotton pellet	5 min	No	CR	NA	NA	Yes	NA
Qudeimat et al. (2007)	NA	NA	Sterile saline irrigation	NA	RMGIC	CR, Amalgam, SSC	NA	NA	In half of them	NA
Suhag et al. (2019)	NA	Irrigation with 2,5% NaOCl	2,5% NaOCl moistened cotton pellets	≤10 min	RMGIC	CR	Postgraduate students	1	Yes	NA
Taha & Khazali (2017)	NA	Irrigation with 2,5% NaOCl	2,5% NaOCl moistened cotton pellets	≤6 min	RMGIC	22/50 Amalgam 27/50 CR	Postgraduate student in endo	1	Yes	NA
Uesrichai et al. (2019)	1−5 mm	10 mL of 2,5% NaOCl	2,5% NaOCl moistened cotton pellets	≤10 min	MTA: RMGIC Biodentine: Nothing	61/69 CR 6/69 SSC	Postgraduate students	10	Yes	NA
Vu et al. (2020)	1.5−2 mm	Irrigation with 2,5% NaOCl	Moist cotton pelletes	2 min	Vitrebond	CR	Endodontist	1	Yes	NA
Wei et al. (2010)	Up to 1 mm	NA	Saline	NA	NA	NA	NA	NA	NA	NA

Abbreviations: CR, composite resin; GIC, glass‐ionomer cement; IRM, intermediate restorative material; NA, not available; RMGIC, resin‐modified glass‐ionomer cement; SSC, stainless steel crown; ZOE, Zinc‐oxide eugenol.

### 3.3 RoB

**Figure 6:**
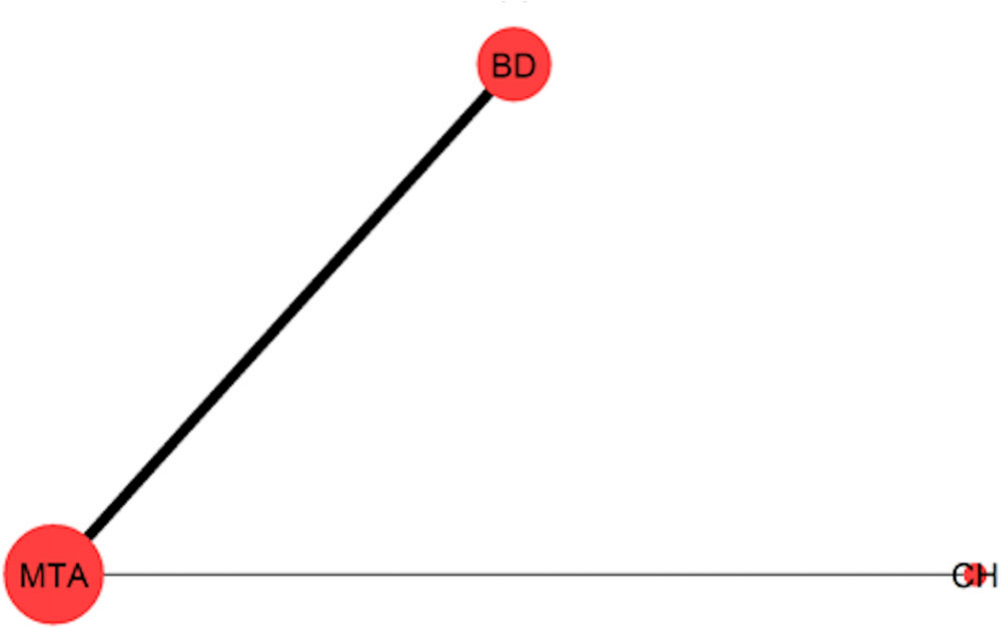
Risk of bias assessment of individual studies.

**Figure 7:**
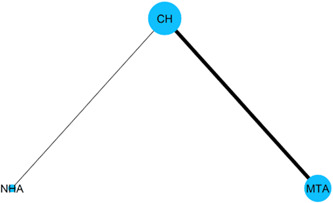
Summary plot of the risk of bias assessment for the included studies.

The RoB assessment for individual studies is presented in Figure 6, and the summary plot of the RoB for the included studies is shown in Figure 7. The most frequent reason threatening the validity of the studies was the lack of blinding of outcome assessors (9/21), and in five (5/21) studies, a significant number of patients were lost to follow‐up.

### 3.4 Transitivity assumption

In the present study, only root maturation and treatment performed were fully observed across the trials. Pulpal diagnosis and “type of teeth included” were missing in 43% and 5% of the trials, respectively. Furthermore, the dominance of comparisons informed by a single trial (three out of five observed comparisons) alongside many categories in pulpal diagnosis complicated the defense of the transitivity assumption (Supporting Information: Figure S1).

### 3.5 Primary outcome

**Figure 8:**
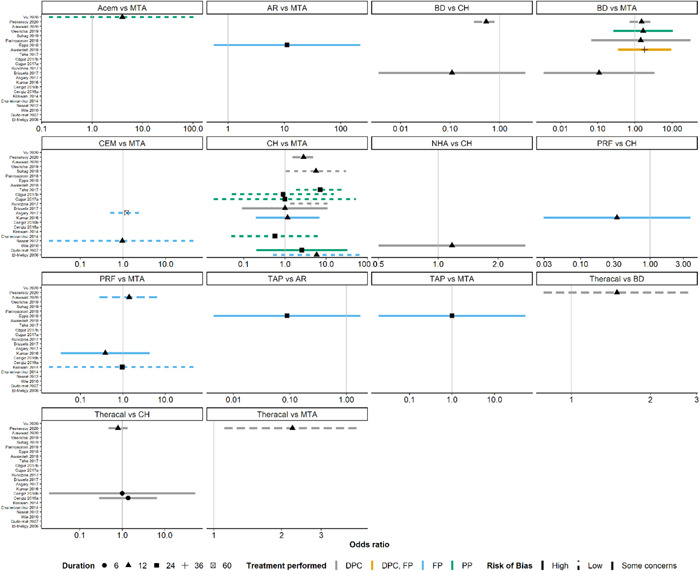
A panel of forest plots on the odds ratio of success (primary outcome) alongside the 95% confidence interval for each trial and corresponding comparison(s). The vertical gray line implies no difference in the compared interventions. The odds ratio above 1 favors the second intervention of the comparison, and the odds ratio below 1 favors the first intervention of the comparison. The risk of bias is indicated with different line shapes, the duration (longest follow‐up duration in months) is indicated with different point shapes, and the treatments performed are indicated with different line colors. DPC, direct pulp capping; FP, full pulpotomy; PP, partial pulpotomy.

Occasioned by the limitations mentioned above, an NMA could not be performed. In the network of interventions for clinical and radiographic success, the OR of success was estimated with considerable uncertainty in most trials as reflected by their 95% CIs that included implausibly low and high values (Figure 8; Supporting Information: Table S2). Consequently, there was weak evidence to support any of the interventions in terms of clinical and radiographic success. An exception was CH versus MTA, where Kundzina et al. (2017) (OR: 4.27, 95% CI: 1.35–13.51), Suhag et al. (Suhag et al., 2019). (OR: 5.62, 95% CI: 1.05–30.17), and Taha and Khazali (Taha & Khazali, 2017) (OR: 7.15, 95% CI: 1.86–27.54) revealed strong evidence in favor of MTA. These trials were judged to have a low RoB. Kundzina et al. (2017) and Suhag et al. (Suhag et al., 2019). performed DPC, and the longest follow‐up for this outcome was 36 and 12 months, respectively. Taha and Khazali (Taha & Khazali, 2017) performed PP and had the longest follow‐up of 24 months for this outcome.

Among the three trials with conclusive results, only Suhag et al. (Suhag et al., 2019). and Taha and Khazali (Taha & Khazali, 2017) reported MOD in at least one arm. In Suhag et al. (Suhag et al., 2019)., the proportion of MOD was 16% and 9% in MTA and CH, respectively. In Taha and Khazali (Taha & Khazali, 2017), the proportion of MOD was 4% and 0% in the corresponding arms. The sensitivity analysis for both trials indicated that the results were robust to different scenarios for the informative missingness OR parameter (robustness index equaled 0.12 and 0 in Suhag et al. (2019) and Taha and Khazali (Taha & Khazali, 2017), respectively).

There was clinical heterogeneity in at least two characteristics in comparison comprising at least two trials and it also manifested as statistical heterogeneity. For instance, the comparison of BD with MTA was informed by five trials that differed in the duration and treatment performed. The two trials on PRF versus MTA (Alawwad et al., 2020; Keswani et al., 2014) provided contradictory evidence, and they also differed in the RoB level. The trials on CH versus MTA exhibited the most considerable heterogeneity in the magnitude, direction, and uncertainty of OR and their characteristics.

The strength of the evidence was not affected by the different ICC values in all pairwise comparisons of the unique cluster trial of Peskersoy et al. (2021) (Supporting Information: Figure S2).

#### 3.5.1 Ad hoc subgroup analysis for CH versus MTA

Subgroup analysis was performed for CH versus MTA, separately, for the treatment performed (i.e., DPC and FP/PP) and root maturation (i.e., mature, immature, and mixed). The choice of the subgroup was predefined in the study protocol where treatment performed (Aguilar & Linsuwanont, 2011) and root development status (Chen et al., 2019) were considered potential effect modifiers.

##### Subgroup analysis for treatment performed

**Figure 9:**
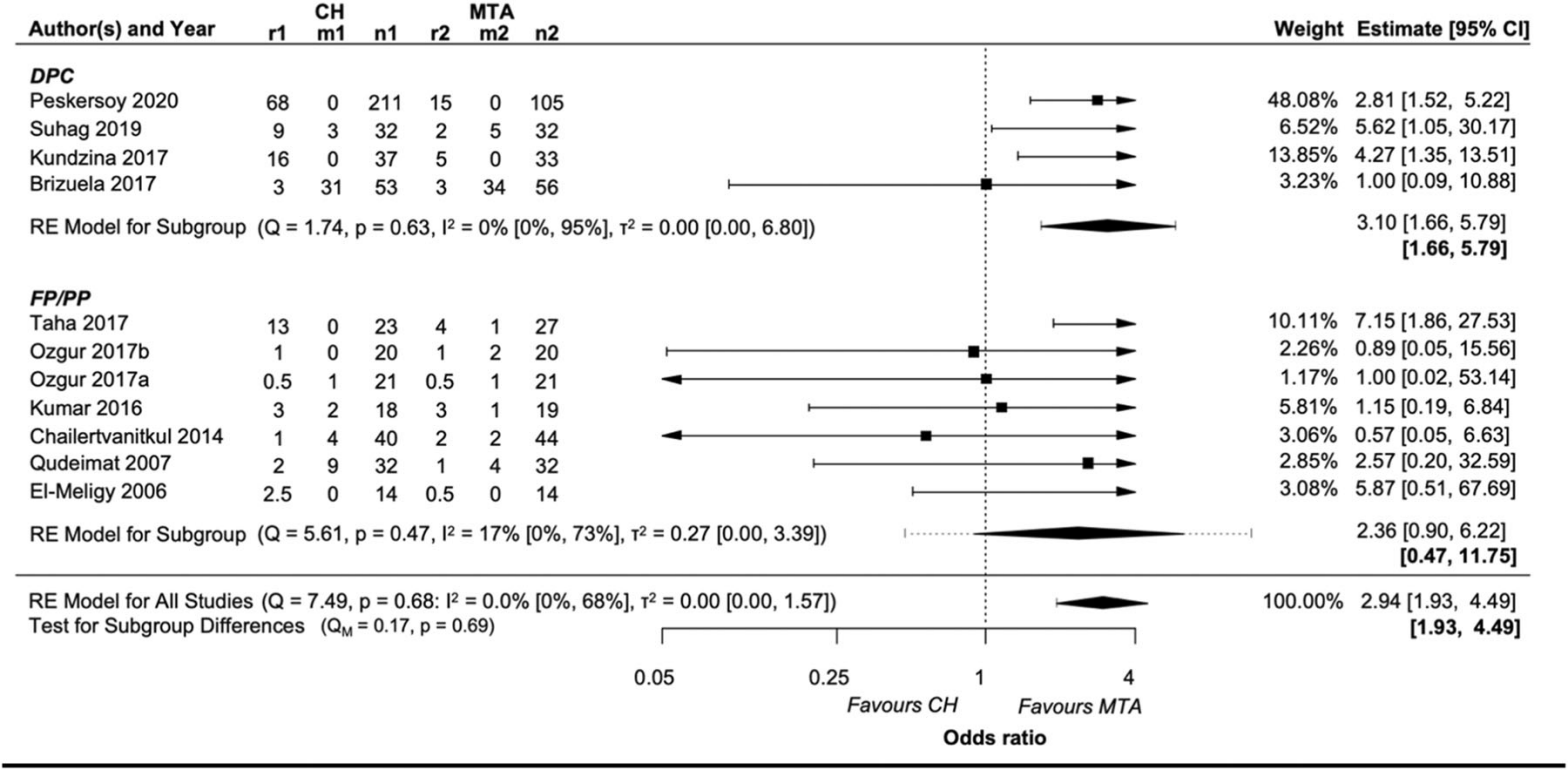
Forest plot of the subgroup analysis in terms of treatment modality (DPC vs Pulpotomy). r1 and r2: number of events (failures) in each group, m1 and m2: number of missing participants in each group, n1 and n2: number of teeth randomized in each group. Summary intervals in bold refer to 95% prediction intervals.

Regardless of the treatment performed, MTA did result in higher treatment success than CH (OR = 2.94, 95% CI: 1.93−4.49), with the largest effect in DPC (OR = 3.10, 95% CI: 1.66−5.79) (Figure 9). There was strong evidence in favor of MTA only for the treatment of DPC. Nevertheless, the wide range of ORs reflected by the corresponding 95% CI lowered our confidence in the results.

##### Subgroup analysis for root maturation

**Figure 10:**
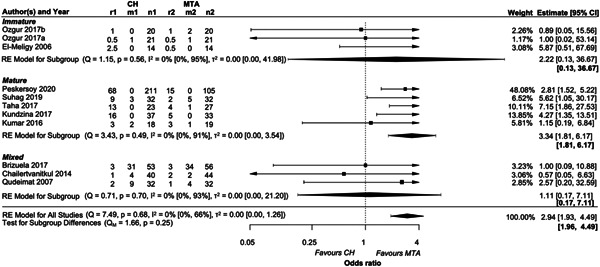
Forest plot of the subgroup analysis in terms of root development status. r1 and r2: number of events (failures) in each group, m1 and m2: number of missing participants in each group, and n1 and n2: number of teeth randomized in each group. Summary intervals in bold refer to 95% prediction intervals.

MTA resulted in higher treatment success than CH regardless of root maturation (OR = 2.94, 95% CI: 1.93−4.49). The largest effect was observed in the subgroup of mature teeth (OR = 3.34, 95% CI: 1.81−6.17), followed by immature (OR = 2.22, 95% CI: 0.13−36.67), and all maturation stages combined (OR = 1.11, 95% CI: 0.17−7.11) (Figure 10). There was strong evidence in favor of MTA only for mature teeth. Nevertheless, the wide range of ORs reflected by the corresponding 95% CI lowers our confidence in the results.

### 3.6 Secondary outcomes

#### 3.6.1 Tooth development

In line with the primary outcome, the OR of tooth development was associated with an implausibly wide 95% CI, and by extent, there was weak evidence to support any of the interventions in the comparisons (Supporting Information: Figure S3; Supporting Information: Table S3). CH with MTA comprised the only informative comparison in the network by three trials. However, the trials differed in various characteristics (treatment performed, follow‐up time, and RoB), and two of them provided contradictory evidence: Qudeimat et al. (2007) favored CH with an OR of 2.01, whereas El‐Meligy and Avery (El‐Meligy & Avery, 2006) favored MTA with a large OR of 5.87.

#### 3.6.2 Bridge formation

Apart from Wei et al. (2010), the trials investigating bridge formation yielded unlikely large ORs as indicated by the width of the corresponding 95% CIs (Supporting Information: Figure S4; Supporting Information: Table S4). Thus, it was not possible to support one material over the other. All four trials comparing CH with MTA used PP and were judged to have a low RoB—apart from Qudeimat et al. (Qudeimat et al., 2007).

#### 3.6.3 Tooth discoloration

The results on tooth discoloration were characterized by implausible values for the OR, rendering their interpretation meaningless. The compared arms were substantially unbalanced regarding the number of discolored teeth, resulting in implausible ORs. Specifically, Awawdeh et al. (2018) and Parinyaprom et al. (2018). reported 27 and 11 cases of discoloration out of the 35 and 31 total for MTA, respectively, and no cases for BD. Taha and Khazali (Taha & Khazali, 2017) reported zero cases in both arms. Uesrichai et al. (2019) reported 28 cases out of 37 for MTA against seven cases out of 32 for BD. All trials were judged to have a high RoB except for Taha and Khazali (2017).

### 3.7 Certainty of evidence

**Figure 11:**
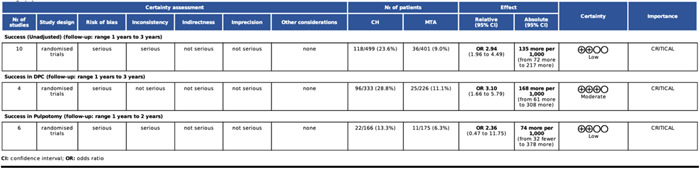
GRADE assessment for the subgroup analysis in terms of treatment performed.

**Figure 12:**
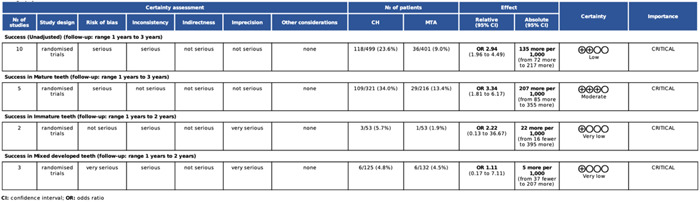
GRADE assessment for the subgroup analysis in terms of root maturation.

Figures 11 and 12 present the GRADE assessment for each subgroup of CH versus MTA. The unadjusted comparison led to serious inconsistency and imprecision and, by that, to a low strength of evidence. The subgroup analysis significantly reduced the heterogeneity, as indicated by the estimated between‐trial standard deviation (τ) in each subgroup. However, τ was accompanied by a substantially wide 95% CI; indicating the presence of substantial statistical heterogeneity in all subgroups. Consequently, the certainty of evidence in the DPC and mature teeth subgroups was found moderate.

## 4 DISCUSSION

The purpose of this study was to uncover the relevant available evidence on the efficacy of capping materials in cases of pulp capping or pulpotomy, and if appropriate, to combine both direct and indirect evidence by means of a NMA. Twenty‐one studies met the inclusion criteria and provided information on the primary outcome of this systematic review comparing 10 different capping agents in total. Combining the information of all available studies using NMA was impossible due to scarcity of the evidence in the networks and difficulty to evaluate the transitivity assumption—the latter resulting from missing data in a large number of categories in the collated characteristics.

CH powder mixed with saline, chemically setting (Dycall) as well as light‐cured CH formulations have been grouped together in the present study. Although differences between pure CH powder/saline mixture and CH cements (like Dycall) have been shown with respect to reparative bridge formation and pulpal inflammation (in favor of pure CH) (Pedano et al., 2020), this evidence is derived from capping non‐carious teeth which have been analyzed in vitro after limited follow‐up periods, or derived indirectly in case of pulpal inflammation. In the present systematic review, the only trial directly comparing Dycall and Calcihyd (Peskersoy et al., 2021) found no significant differences in VPT success rate between both groups. Besides that, we could not find any trial comparing different CH formulations in the capping of cariously exposed pulps. While these do not represent identical substances, they share the biological mechanism of action in vital pulp therapy by releasing hydroxyl ions and creating an alkaline environment, and therefore they were grouped together.

It was chosen to analyze FP and PP in the same subgroup. The rationale behind this decision was first conceptual as FP and PP represent different values in the same continuum and secondary it was impossible to draw lines between the two entities both between as within studies in terms of definition as well as the exact intervention performed. DPC on the contrary does not involve any pulp amputation, and thus, it was analyzed separately. The differences between the procedures were not the main focus of our study. Therefore, presenting them separately in the subgroup analysis could have unnecessarily complicated the interpretation of findings.

Some intransitivity in the collated evidence base may be expected. Intransitivity may manifest as statistical heterogeneity or inconsistency between the direct and indirect effects of a given comparison (Salanti, 2012). In this case, a meta‐regression analysis can be implemented to adjust for intransitivity on the treatment effects, even partially. A fully observed covariate, at least for most of the observed comparisons, and a sufficient number of trials per comparison are prerequisites for the successful application of such analysis. In our study, only half the effect modifiers were fully observed to allow for the evaluation of the transitivity assumption. At first, we attempted to perform NMA for the primary outcome despite the scarce evidence and the difficulty to defend the transitivity assumption. However, the model failed to converge due to zero events observed in two trials (see “Analyses planned and those performed” in Supporting Information Material). The convergence issue was overcome after assigning a weakly informative prior distribution on the summary log OR for comparisons with the reference intervention (MTA). However, the posterior standard deviation of the summary log OR for comparisons with MTA was substantial. Hence, we decided to abstain from NMA for all outcomes.

The comparison of CH with MTA was the most informative in the network with 11 studies contributing data. However, overlooking the substantial heterogeneity in the included trials and pooling them in a random‐effects pairwise meta‐analysis would have led to invalid results. A subsequent meta‐regression analysis would require many more trials to accommodate all three characteristics reported in the protocol (RoB, treatment performed, and root maturity) and explain a substantial part of the variability observed in the results of the included trials. Therefore, we performed a subgroup analysis in terms of the type of vital pulp therapy (DPC vs. Pulpotomy) and root maturity. When all studies were pooled together, MTA outperformed CH in terms of clinical and radiographic success. The subgroup analysis though indicated strong evidence of that superiority only in DPC and mature teeth subgroups. This difference among the subgroups might be attributed to various reasons. In the case of DPC, this may derive from inherent inferiority (lower success rate) compared to pulpotomy procedures (Aguilar & Linsuwanont, 2011). The interface between pulp and capping material is closer to the carious exposure site in DPC than in partial or full pulpotomy. This predisposes to an increased likelihood of contamination. Possibly MTA better inactivates remaining contamination or irritating substances due to more intense calcium and hydroxyl ion release (Natale et al., 2015), better adhesion to dentin, and better mechanical properties. Furthermore, detection bias might be encountered more frequently in the pulpotomy cases rather than DPC, as the cold test has lower sensitivity due to the deeper placement of pulpal tissues in the first case. It is, thus, not only that the number of failed cases that might be higher in the DPC subgroup but also a bigger percentage of them get detected contrary to the pulpotomy counterpart. Immature teeth, on the other hand, have greater regenerative capacity and might therefore be less dependent on the material properties of the capping agent. An alternative explanation for the second subgroup might be a prognostic imbalance between children and adults. It has to be stressed that these explanations remain speculative, as there is no conclusive evidence for any.

The findings of the present study are in accordance with the studies of Cushley et al. (Cushley et al., 2021) and Li et al. (Li et al., 2019) that draw similar conclusions. Minor differences are observed as Cushley et al. (Cushley et al., 2021) did not include two studies (Brizuela et al., 2017; Peskersoy et al., 2021), and by that, the effect was estimated with less precision, including the value of no difference in the corresponding 95% CI. Li et al. (2019)., on the other hand, did not include the split‐mouth study of El‐Meligy and Avery (El‐Meligy & Avery, 2006) and considered all missing participants as failures. Furthermore, they performed a fixed‐effect meta‐analysis, while we used random‐effect subgroup analysis, which accounts for the variation in the trial results due to the inherent clinical and methodological heterogeneity. The application of the fixed‐effect model may explain the statistically significant results obtained in their analysis, as this model discounts statistical heterogeneity, and hence, leads to spuriously more precise (narrower CI) results. Regardless of the minor discrepancies in precision and statistical significance, both the aforementioned SRs and our study favored MTA with a comparable effect size (ORs were very close to each other).

The substantial heterogeneity of the trials for the remaining comparisons did not allow any meta‐analysis. In Figure 8, we can see that MTA performed comparably to Biodentine, CEM, PRF, and TAP in terms of the primary outcome; a slightly lower number of failures was observed with MTA when compared with Theracal, AR, and Acem. Likewise, Biodentine seemed to perform better than both CH and Theracal.

Discoloration is an important patient‐centered outcome (Williamson et al., 2017) that may impact the aesthetics of the patient. Although tooth discoloration after using pulp capping agents has been frequently reported (Parirokh & Torabinejad, 2010), only four studies (Awawdeh et al., 2018; Parinyaprom et al., 2018; Taha & Khazali, 2017; Uesrichai et al., 2019) recorded data on discoloration. In an attempt to reduce observer bias, clinical pictures of the teeth were blindly assessed in two studies (Parinyaprom et al., 2018; Uesrichai et al., 2019). No information about the discoloration assessment method was reported in the other two studies. Consistently, more cases of discoloration were observed in the MTA group when the latter was compared with Biodentine, while Taha and Khazali (Taha & Khazali, 2017) did not report any discoloration when MTA was compared with CH. The results in the comparison of Biodentine versus MTA were consistent with the evidence available in the literature (Mozynska et al., 2017); the magnitude of the effect reported, though, especially by Awawdeh et al. (2018) is clinically implausible (Pereira et al., 2012).

Another important endpoint after vital pulp therapy is root development in the case of immature teeth (Chen et al., 2019). Similarly, implausibly wide CI was reported in the studies investigating this outcome, as in all cases, the line of no difference was included in the 95% CI. Moreover, in many comparisons, the results were clinically implausibly, hence, reducing our confidence to indicate one material was better than another.

Data about bridge formation were very scarce as well. Considerable imprecision was observed once again, while no apparent benefit was observed between the different materials. An outcome that was not reported in any of the included studies was the cost‐effectiveness of the materials.

One of the important things in vital pulp therapy is the adoption of an optimized protocol (Duncan et al., 2019), including strict aseptic measures, the use of magnification, and practitioner experience. Although the use of RD before the initiation of the treatment was reported in almost every study, preoperative disinfection of the operating field was reported only in seven studies (Asgary et al., 2017; Awawdeh et al., 2018; Brizuela et al., 2017; Nosrat et al., 2013; Suhag et al., 2019; Taha & Khazali, 2017; Vu et al., 2020). Similarly, the disinfection of the exposed pulp was reported in half of the studies (Table 4); the use of sterile water, NaOCl, and Er; Cr:YSGG laser irradiation were the methods of exposure cleaning.

A major threat to the external validity of the present study was that in only one primary study (Brizuela et al., 2017), the use of magnification (loupes x3,5) was reported. As suggested by ESE (Duncan et al., 2019), the use of magnification is crucial in VPT involving carious exposure to enhance the operative protocol. It is, thus, questionable whether the results of the present study are applicable to a general practitioner or an endodontist using either loupes or an operating microscope. Future trialists should embrace the clinical protocol recommendations from ESE (Duncan et al., 2019) and report their studies accordingly.

The RoB assessment for the primary outcome indicated that almost half of the studies posed a threat to the validity of the findings. The most common type of bias was either observer or attrition bias. In many studies, the outcome assessor was not blinded, and as such, the decision reached could be flawed (Schulz & Grimes, 2002). In five studies, a considerable percentage of participants (more than 20%) was missing, which also may impact the outcome estimation (Spineli et al., 2015); although the sensitivity analysis revealed that the primary analysis results were robust to different assumptions about the missingness mechanism in the compared interventions.

Many of the materials compared in the included studies were examined for the first time, and thus, the comparisons in the network were informed by a single trial. Many of these agents such as nano‐hydroxyapatite, Accemanan, Abscess Remedy, and CEM are not readily available in the market, while the TAP and PRF require considerable effort to prepare. MTA, Biodentine, CH, and Theracal represent more clinically relevant options for the clinician as they are commercially available, and preparation and application are relatively easy. In a future update of the present study, we intend to exclude interventions that do not present clinical interest in terms of availability and ease of use.

The present study included mostly small trials with a small number of events. Therefore, it was difficult to defend the assumption of approximate normality, which raises the risk of biased and spuriously precise estimations (Jackson & White, 2018). In the present study, the total sample size ranged from 26 to 169 (median: 60 participants) in 20 trials; Asgary et al. (Asgary et al., 2017) randomized 413 participants in CEM versus MTA, and the cluster‐RCT of Peskersoy et al. (Peskersoy et al., 2021). randomized 226 participants. Furthermore, the risk of events (i.e., the proportion of observed events in the completers group) ranged from 0.00 to 0.56 (median: 0.07) across the arms of these trials. Small study effects (Sterne et al., 2000) could not be easily detected by any of the commonly used methods (Sterne et al., 2011) due to the low number of studies in the meta‐analysis.

For future studies in this area, researchers should try to design larger‐scale, better‐quality RCTs adhering to the protocol suggested by ESE and also take into consideration patient‐related outcomes such as discoloration and cost‐effectiveness.

## 5 CONCLUSION

Considerable clinical and statistical heterogeneity among the trials did not allow NMA. The ad hoc subgroup analysis indicated that the clinical and radiographic success of MTA was higher than that of CH but only in mature teeth and DPC cases where the strength of evidence was moderate.

## AUTHOR CONTRIBUTIONS

All authors were involved with the protocol of the study and the preparation and review of the manuscript. Athanasios Fasoulas, Georgios Keratiotis, and Maarten A. Meire carried out electronic search, screening, data extraction, and RoB and GRADE assesment. Loukia Spineli performed statistical analysis.

## CONFLICT OF INTEREST STATEMENT

The authors declare no conflict of interest.

## Supporting information

Supporting information.

Supporting information.

Supporting information.

Supporting information.

Supporting information.

## Data Availability

The datasheets of the study are available after communication with the corresponding author.
