# Supplementary material for: Comparative efficacy of materials used in patients undergoing pulpotomy or direct pulp capping in carious teeth: A systematic review and meta‐analysis
Source: Clin Exp Dent Res. 2023 Sep 14;9(6):1129–48. doi: 10.1002/cre2.767 (PMC10728530; doi:10.1002/cre2.767)
Supplement: Supplementary file 1 — Supporting information. [file CRE2-9-1129-s004.docx]

# Methods section

***Modelling missing participant outcome data***

To account for missing participant outcome data (MOD), we employed the pattern-mixture model in each arm of every trial. This model distinguishes between the missing participants and those completing the assigned arm (called ‘completers’). The pattern-mixture model is advantageous to the exclusion and imputation of MOD for maintaining the randomised sample and accounting for the uncertainty induced by MOD in the estimated treatment effects. We used the informative missingness odds ratio (IMOR) parameter to quantify the departure from the missing at random assumption (MAR) (1). This parameter is defined as the odds of experiencing the event given the missing participants to the odds of experiencing the event given the completers. We assumed the IMOR parameter to be normally distributed in the logarithmic scale for each trial’s arm like with the OR. The mean of the normal distribution indicates the assumed missingness mechanism on average, and the variance reflects our uncertainty about this assumption. The reasons behind MOD are practically unknown if missing participants have not been followed up. Following the relevant recommendations, we considered the MAR assumption to be the starting point for all trial arms (2). Specifically, we set the mean equal to 0 and the variance equal to 1, which corresponds to the 95% range [-1.96, 1.96] of log IMOR values, or equivalently to the 95% range [0.14, 7.10] of IMOR values. The R code to apply the aforementioned pattern-mixture model in every trial can be found in the appendix (Additional file 2) of the article (3).

For those trials providing conclusive evidence for the investigated outcomes (i.e. the 95% CI did not include the value of no difference) after adjusting for MOD, we performed a sensitivity analysis using the following informative scenarios for log IMOR: log IMOR $\in\left\{ -log\left( 3 \right),-log\left( 2 \right),log\left( 2 \right),log\left( 3 \right) \right\}$ We used the recently proposed robustness index to infer the robustness of the primary analysis results in each trial (4). This index measures the overall departure of the sensitivity analyses from the primary analyses results, and it is measured in the log OR scale. We chose $\log\left( 1.32 \right)$ to be the robustness threshold, following the relevant recommendations (4). A robustness index of at least $\log\left( 1.32 \right)$ implies a lack of robustness in the primary analysis results of the trial. The R code to calculate the robustness index is publicly available at [https://github.com/ LoukiaSpin/Quantifying-Robustness-in-Meta-analysis.git](https://github.com/%20LoukiaSpin/Quantifying-Robustness-in-Meta-analysis.git).

References

1. White IR, Higgins JP, Wood AM. Allowing for uncertainty due to missing data in meta-analysis--part 1: two-stage methods. Stat Med. 2008;27(5):711-727.
2. Higgins JP, White IR, Wood AM. Imputation methods for missing outcome data in meta-analysis of clinical trials. Clin Trials. 2008;5(3):225-239.
3. Spineli LM, Kalyvas C. Comparison of exclusion, imputation and modelling of missing binary outcome data in frequentist network meta-analysis. BMC Med Res Methodol. 2020;20(1):48.
4. Spineli LM, Kalyvas C, Papadimitropoulou K. Quantifying the robustness of primary analysis results: A case study on missing outcome data in pairwise and network meta-analysis. Res Synth Methods. 2021;12(4):475-490.

#

# Analyses planned and those performed

Initially, we considered applying Bayesian network meta-analysis to obtain the log odds ratio (OR) using for all outcomes the binomial distribution, which is the exact distribution of the binary outcome data. Nevertheless, convergence failed for the two-arm trial of Vu et al. (2020; they compared Acem with MTA) and the three-arm trial of Eppa et al. (2018; they included TAP, AR and MTA). Both trials had zero events in at least one arm. Overall, eight out of 21 trials (38%) reported zero events in at least one arm: three had zero events in one arm, and five had zero events in both arms. We assigned a normal prior distribution with zero mean and standard deviation 2.82 on the summary log OR for comparisons with the reference intervention (MTA) to overcome the convergence issue. Günhan et al. (1) recommended this weakly-informative prior for random-effects pairwise meta-analysis of few trials with rare events. Convergence was successful for all model parameters; however, substantial uncertainty in the estimation of the log OR remained for the comparisons above: the posterior standard deviation of log OR ranging from 1.26 to 2.24. By including the consistency equation, this considerable uncertainty affected the estimation of many comparisons in the network. Since the posterior standard deviation of log OR ranged from 0.38 to 3.00, it led to 95% credible intervals that included implausible low and large values of log OR.

Eventually, we decided to abstain from synthesising the trials and using Bayesian methods. Therefore, we resorted to estimating the trial-specific log OR and standard error using a large-sample Taylor series approximation following White et al. (2).

References

1. Günhan BK, Röver C, Friede T. Random-effects meta-analysis of few studies involving rare events. Res Synth Methods. 2020;11(1):74-90.
2. White IR, Higgins JP, Wood AM. Allowing for uncertainty due to missing data in meta-analysis--part 1: two-stage methods. Stat Med. 2008;27(5):711-727.

***Figures:***


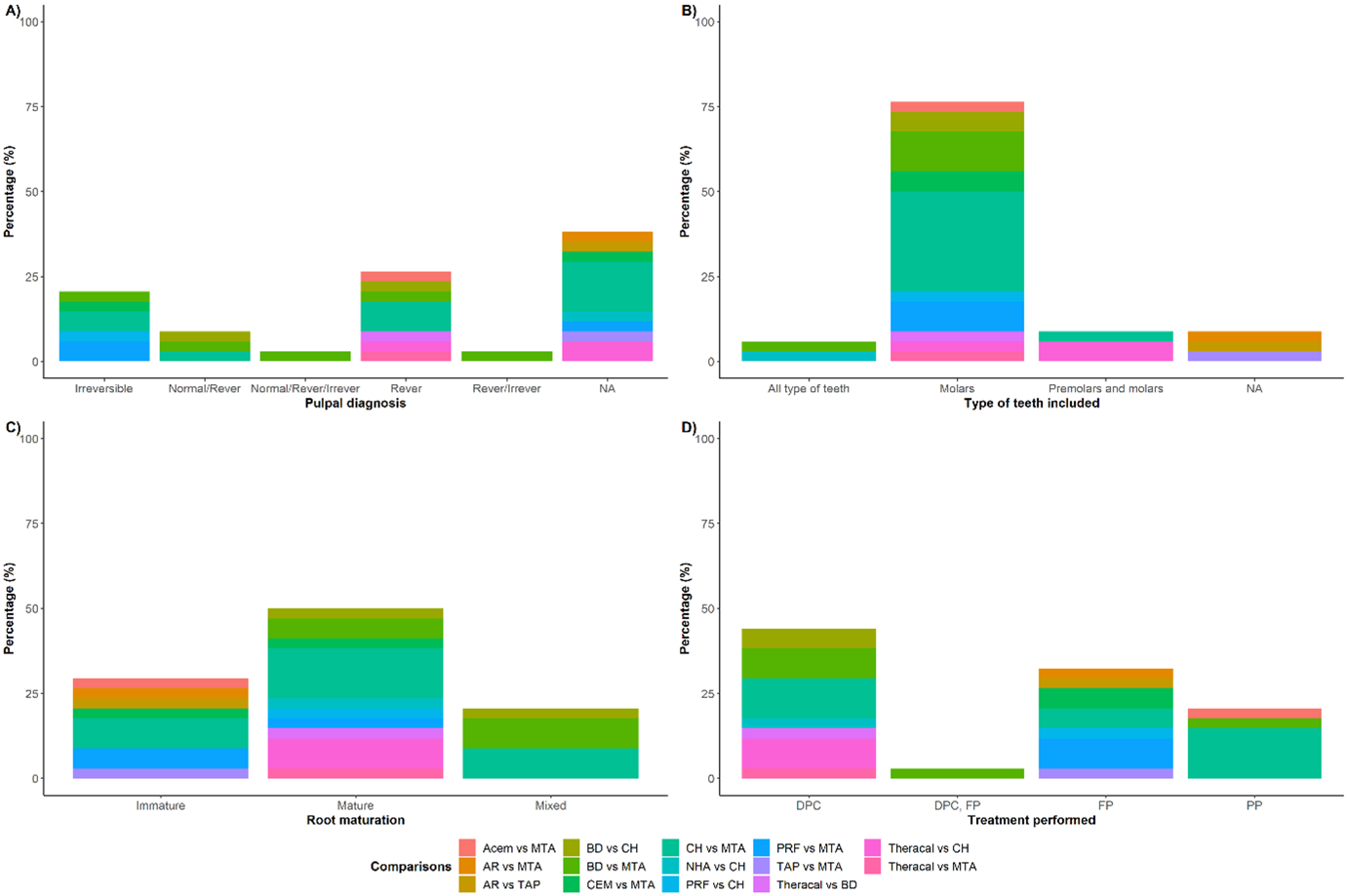


**Figure S1.** Stacked barplots for the evaluation of the transitivity assumption (A – D). The different colours in each bar refer to different observed pairwise comparisons as indicated in the legend ‘Comparisons’. The percentage in the y-axis refers to the percentage of trials investigating a pairwise comparison (as indicated by the corresponding colour).


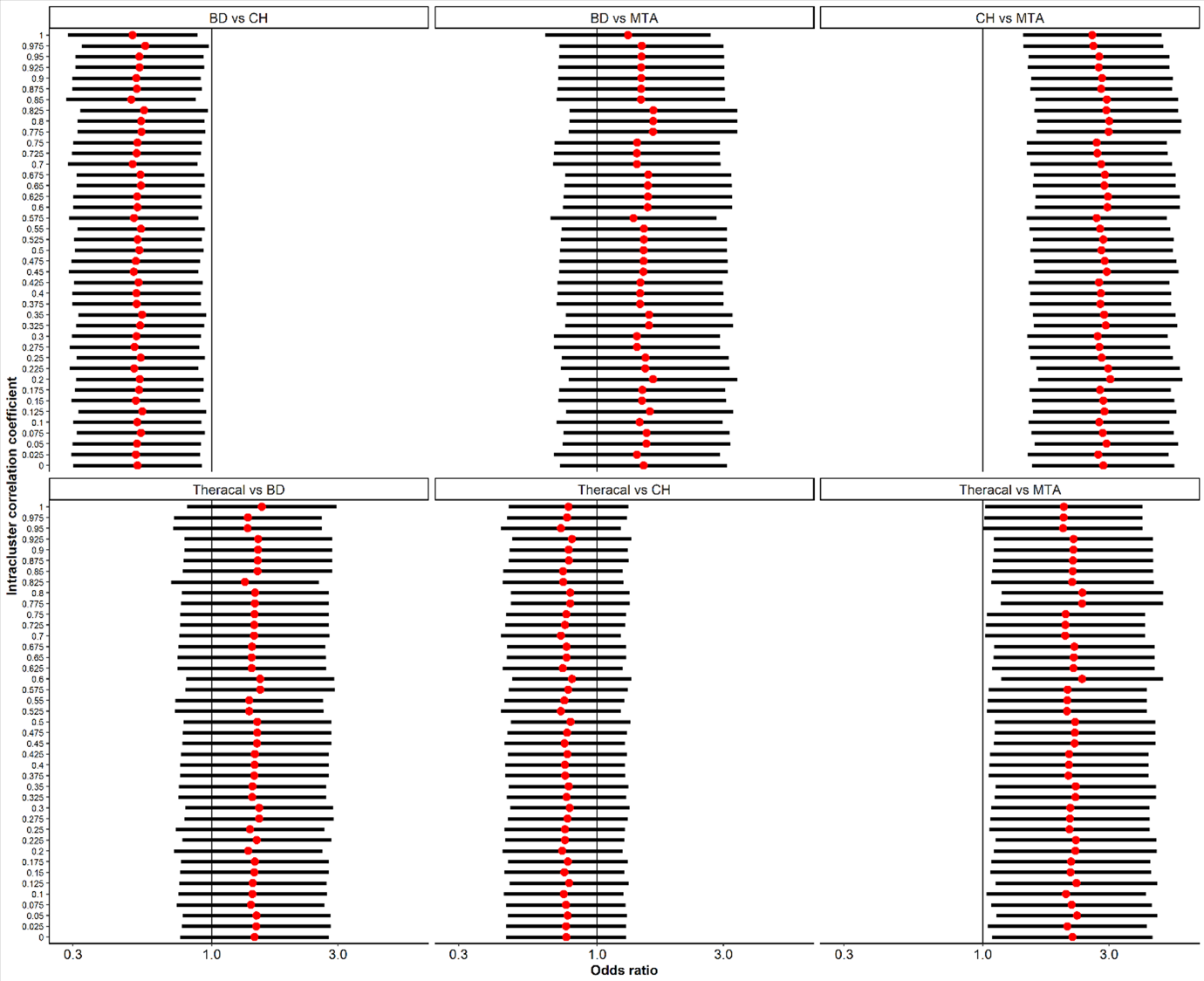


**Figure S2.** The odds ratio (OR) and 95% confidence interval (CI) for different values of the intracluster correlation coefficient (ICC). Results are illustrated for each possible comparison of the four-arm trial of Peskersoy et al. (2020). The y-axis refers to the positive range of the ICC values. Lines refer to the 95% CI and red points refer to the estimated OR. Lines that cross the vertical line of no difference imply inconclusive results, and lines that do not cross the vertical line of no difference imply conclusive results.


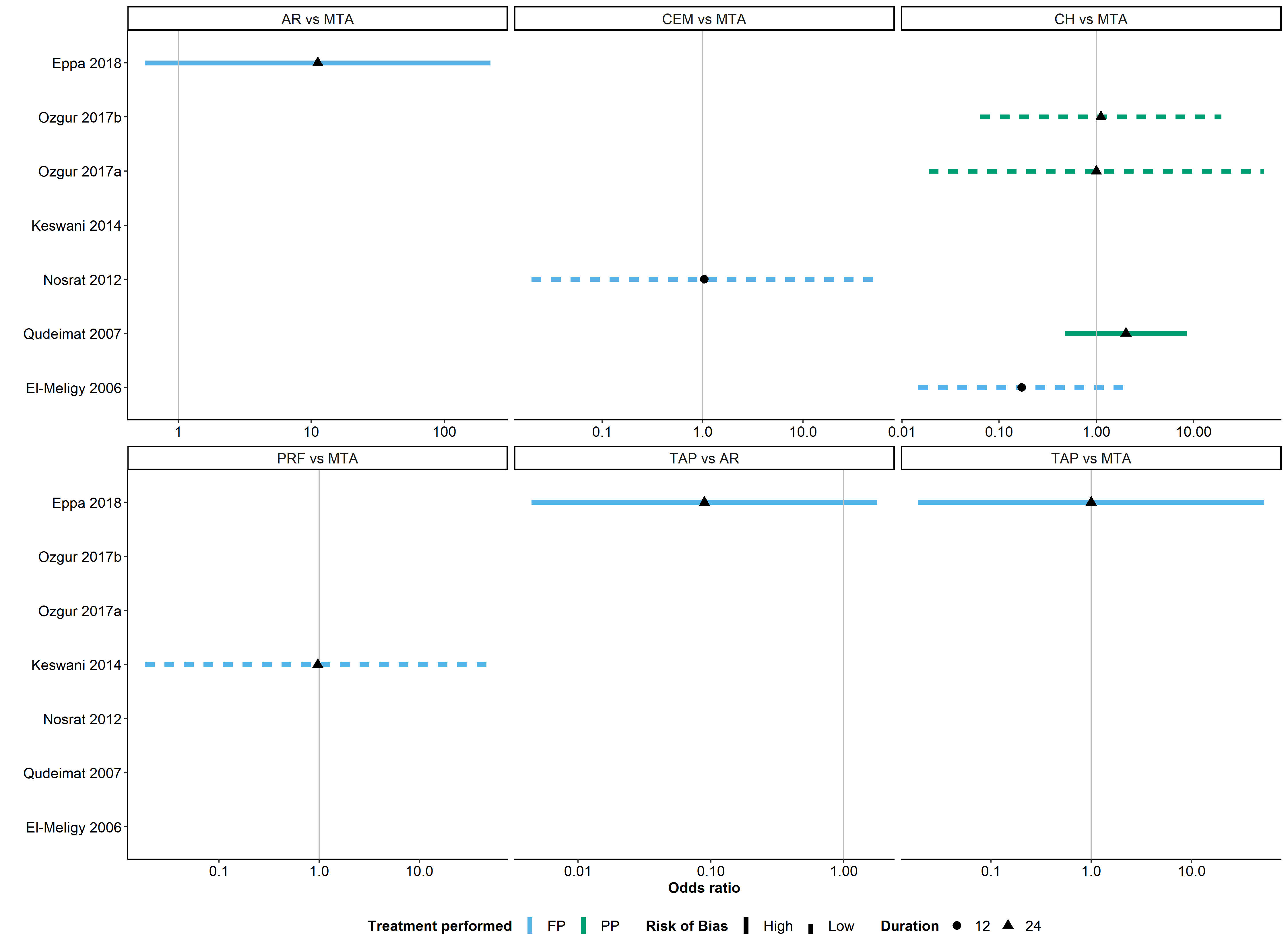


**Figure S3.** Forest plots of all different comparisons in terms root development across the studies. DPC: Direct pulp capping, FP: Full pulpotomy
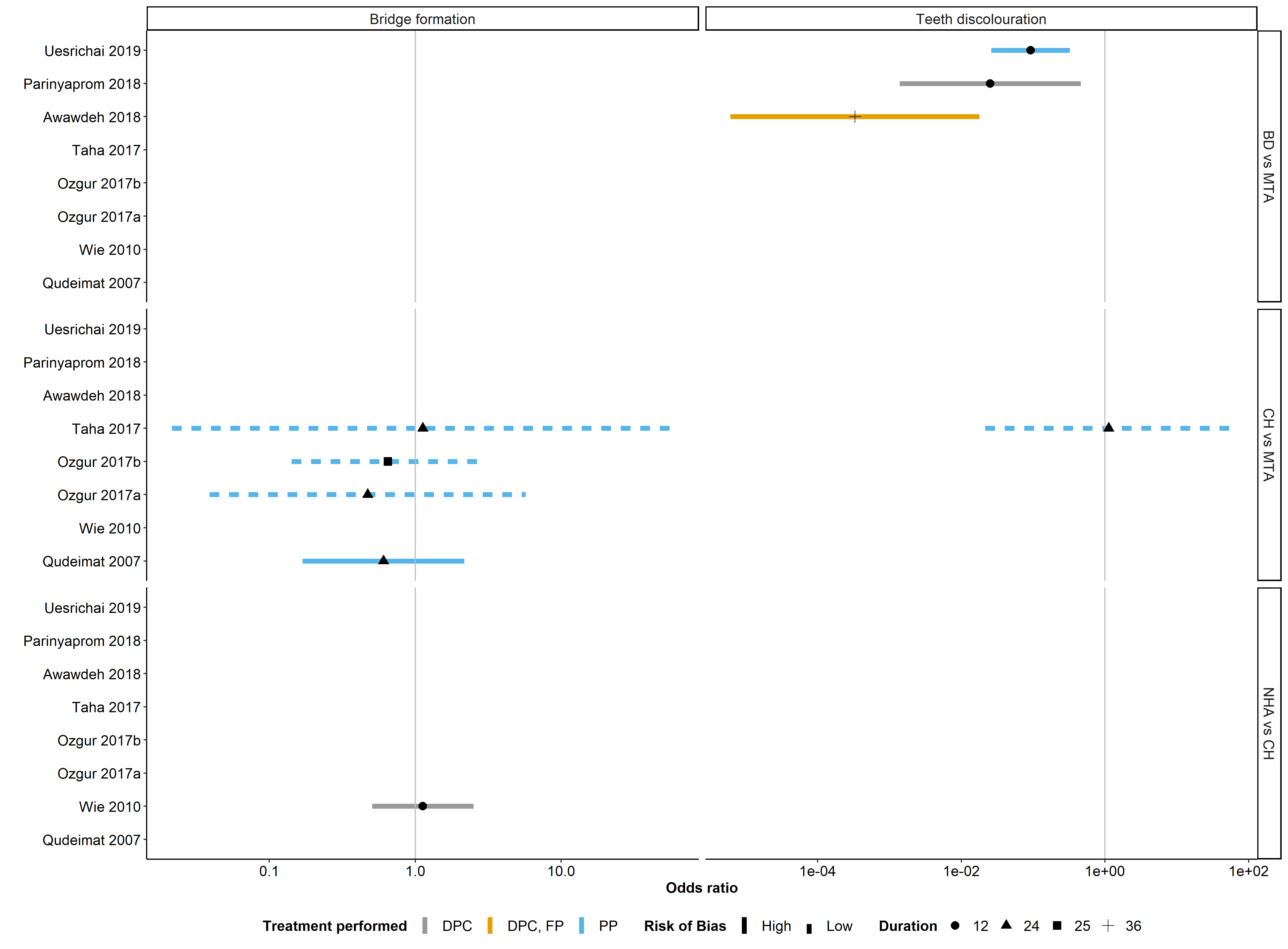


**Figure S4.** Forest plots of all different comparisons in terms discoloration and bridge formation across the studies. DPC: Direct pulp capping, FP: Full pulpotomy, PP: Partial pulpotomy
