# Supplementary material for: Comparative efficacy of materials used in patients undergoing pulpotomy or direct pulp capping in carious teeth: A systematic review and meta‐analysis
Source: Clin Exp Dent Res. 2023 Sep 14;9(6):1129–48. doi: 10.1002/cre2.767 (PMC10728530; doi:10.1002/cre2.767)
Supplement: Supplementary file 3 — Supporting information. [file CRE2-9-1129-s003.docx]

| **Trial** | **Comparison** | **OR** | **95% CI** | | **Performance** | **RoB** | **Duration (Months)** |
| --- | --- | --- | --- | --- | --- | --- | --- |
| Alawwad 2020 | PRF vs MTA | 1.40 | 0.28 | 7.01 | FP | Some concerns | 12 |
| Peskersoy 2020 | Theracal vs CH | 0.79 | 0.47 | 1.33 | DPC | Some concerns | 12 |
| Peskersoy 2020 | BD vs CH | 0.53 | 0.30 | 0.93 | DPC | Some concerns | 12 |
| Peskersoy 2020 | CH vs MTA | 2.81 | 1.52 | 5.22 | DPC | Some concerns | 12 |
| Peskersoy 2020 | Theracal vs BD | 1.49 | 0.78 | 2.84 | DPC | Some concerns | 12 |
| Peskersoy 2020 | Theracal vs MTA | 2.24 | 1.11 | 4.48 | DPC | Some concerns | 12 |
| Peskersoy 2020 | BD vs MTA | 1.50 | 0.73 | 3.10 | DPC | Some concerns | 12 |
| Vu 2020 | Acem vs MTA | 3.95 | 0.14 | 112.24 | PP | Low | 12 |
| Suhag 2019 | CH vs MTA | 5.62 | 1.05 | 30.17 | DPC | Low | 12 |
| Uesrichai 2019 | BD vs MTA | 1.67 | 0.26 | 10.54 | PP | High | 12 |
| Awawdeh 2018 | BD vs MTA | 1.82 | 0.35 | 9.43 | DPC, FP | High | 36 |
| Eppa 2018 | TAP vs MTA | 1.01 | 0.02 | 52.83 | FP | High | 24 |
| Eppa 2018 | AR vs MTA | 11.18 | 0.56 | 222.94 | FP | High | 24 |
| Eppa 2018 | TAP vs AR | 0.09 | 0.01 | 1.78 | FP | High | 24 |
| Parinyaprom 2018 | BD vs MTA | 1.43 | 0.07 | 30.58 | DPC | High | 12 |
| Asgary 2017 | CEM vs MTA | 1.23 | 0.50 | 2.99 | FP | Low | 60 |
| Brizuela 2017 | CH vs MTA | 1.01 | 0.09 | 10.88 | DPC | High | 12 |
| Brizuela 2017 | BD vs CH | 0.11 | 0.01 | 3.32 | DPC | High | 12 |
| Brizuela 2017 | BD vs MTA | 0.11 | 0.01 | 3.37 | DPC | High | 12 |
| Kundzina 2017 | CH vs MTA | 4.27 | 1.35 | 13.51 | DPC | Low | 36 |
| Ozgur 2017b | CH vs MTA | 0.89 | 0.05 | 15.56 | PP | Low | 24 |
| Ozgur 2017a | CH vs MTA | 1.01 | 0.02 | 53.14 | PP | Low | 24 |
| Taha 2017 | CH vs MTA | 7.15 | 1.86 | 27.54 | PP | Low | 24 |
| Cengiz 2016b | Theracal vs CH | 1.01 | 0.02 | 53.66 | DPC | High | 6 |
| Cengiz 2016a | Theracal vs CH | 1.37 | 0.29 | 6.61 | DPC | High | 6 |
| Kumar 2016 | CH vs MTA | 1.15 | 0.19 | 6.84 | FP | High | 12 |
| Kumar 2016 | PRF vs CH | 0.33 | 0.03 | 3.76 | FP | High | 12 |
| Kumar 2016 | PRF vs MTA | 0.38 | 0.03 | 4.28 | FP | High | 12 |
| Chailertvanitkul 2014 | CH vs MTA | 0.57 | 0.05 | 6.63 | PP | Low | 24 |
| Keswani 2014 | PRF vs MTA | 0.96 | 0.02 | 51.31 | FP | Low | 24 |
| Nosrat 2012 | CEM vs MTA | 0.96 | 0.02 | 50.46 | FP | Low | 12 |
| Wie 2010 | NHA vs CH | 1.17 | 0.50 | 2.74 | DPC | High | 12 |
| Qudeimat 2007 | CH vs MTA | 2.57 | 0.20 | 32.60 | PP | High | 24 |
| El-Meligy 2006 | CH vs MTA | 5.87 | 0.51 | 67.69 | FP | Low | 12 |

**Table S2.** Primary outcome: number of failures
