# Supplementary material for: Comparative efficacy of materials used in patients undergoing pulpotomy or direct pulp capping in carious teeth: A systematic review and meta‐analysis
Source: Clin Exp Dent Res. 2023 Sep 14;9(6):1129–48. doi: 10.1002/cre2.767 (PMC10728530; doi:10.1002/cre2.767)
Supplement: Supplementary file 4 — Supporting information. [file CRE2-9-1129-s001.docx]

| **Trial** | **Comparison** | **OR** | **95% CI** | | **Treatment** | **RoB** | **Duration (months)** |
| --- | --- | --- | --- | --- | --- | --- | --- |
| Eppa 2018 | AR vs MTA | 11.18 | 0.56 | 222.94 | FP | High | 24 |
| Eppa 2018 | TAP vs AR | 0.09 | 0.01 | 1.78 | FP | High | 24 |
| Eppa 2018 | TAP vs MTA | 1.00 | 0.02 | 52.83 | FP | High | 24 |
| Ozgur 2017b | CH vs MTA | 1.12 | 0.06 | 19.43 | PP | Low | 24 |
| Ozgur 2017a | CH vs MTA | 1.01 | 0.02 | 53.14 | PP | Low | 24 |
| Keswani 2014 | PRF vs MTA | 0.96 | 0.02 | 51.31 | FP | Low | 24 |
| Nosrat 2012 | CEM vs MTA | 1.04 | 0.02 | 54.66 | FP | Low | 12 |
| Qudeimat 2007 | CH vs MTA | 2.01 | 0.47 | 8.53 | PP | High | 24 |
| El-Meligy 2006 | CH vs MTA | 0.17 | 0.01 | 1.96 | FP | Low | 12 |

**Table S3.** Secondary outcome: tooth development
