# Supplementary material for: Comparative efficacy of materials used in patients undergoing pulpotomy or direct pulp capping in carious teeth: A systematic review and meta‐analysis
Source: Clin Exp Dent Res. 2023 Sep 14;9(6):1129–48. doi: 10.1002/cre2.767 (PMC10728530; doi:10.1002/cre2.767)
Supplement: Supplementary file 5 — Supporting information. [file CRE2-9-1129-s005.docx]

| **Trial** | **Comparison** | **OR** | **95% CI** | | **Performance** | **RoB** | **Duration (Months)** |
| --- | --- | --- | --- | --- | --- | --- | --- |
| *Teeth discolouration* | | | | | | | |
| Uesrichai 2019 | BD vs MTA | 0.09 | 0.03 | 0.32 | PP | High | 12 |
| Awawdeh 2018 | BD vs MTA | 0.01 | 0.01 | 0.02 | DPC, FP | High | 36 |
| Parinyaprom 2018 | BD vs MTA | 0.03 | 0.01 | 0.46 | DPC | High | 12 |
| Taha 2017 | CH vs MTA | 1.13 | 0.02 | 59.1 | PP | Low | 24 |
| *Bridge formation* | | | | | | | |
| Ozgur 2017b | CH vs MTA | 0.65 | 0.14 | 2.96 | PP | Low | 25 |
| Ozgur 2017a | CH vs MTA | 0.47 | 0.04 | 5.73 | PP | Low | 24 |
| Taha 2017 | CH vs MTA | 1.13 | 0.02 | 59.1 | PP | Low | 24 |
| Wie 2010 | NHA vs CH | 1.13 | 0.51 | 2.51 | DPC | High | 12 |
| Qudeimat 2007 | CH vs MTA | 0.61 | 0.17 | 2.17 | PP | High | 24 |

**Table S4.** Secondary outcomes: teeth discolouration and bridge formation
